# Survival and inactivation of human norovirus GII.4 Sydney on commonly touched airplane cabin surfaces

**DOI:** 10.3934/publichealth.2020046

**Published:** 2020-07-29

**Authors:** Dorra Djebbi-Simmons, Mohammed Alhejaili, Marlene Janes, Joan King, Wenqing Xu

**Affiliations:** School of Nutrition and Food Sciences, Louisiana State University Agricultural Center, Baton Rouge, LA, USA

**Keywords:** human norovirus, airplane cabin surfaces, survival, inactivation, EPA registered disinfectants

## Abstract

Human norovirus (HuNoV) is one of the leading causes of acute gastroenteritis globally. HuNoV outbreaks have been recently reported during air travels. Contaminated surfaces are known as a critical transmission route at various settings. The aim of this study was to provide key information about the survival and the decontamination of HuNoV on three commonly touched airplane cabin surfaces.

In this study, we monitored the survival of HuNoV on seat leather, plastic tray table, and seatbelt for 30 days, with and without additional organic load (simulated gastric fluid). The efficacy of two EPA registered anti-norovirus disinfectants were also evaluated. Results showed that HuNoV was detected at high titers (>4 log_10_ genomic copy number) for up to 30 days when additional organic load was present. Both tested disinfectants were found highly ineffective against HuNoV when the surface was soiled.

The study showed that when the organic load was present, HuNoV was highly stable and resistant against disinfectants. Findings from this study indicated that appropriate procedures should be developed by airline companies with the help of public health authorities to decrease passengers' exposure risk to HuNoV.

## 1. Introduction

Human norovirus (HuNoV) is the most common cause of acute gastroenteritis worldwide where norovirus genogroup II, genotype4 (GII.4) accounts for 60–90% of all HuNoV gastroenteritis illnesses annually [1]. HuNoV belongs to the family of the *Caliciviridae*, non-enveloped, and genetically diverse positive-sense single stranded RNA viruses [2]. The main transmission route of HuNoV is through direct contact with a sick person and/or their infected gastrointestinal bodily fluids (vomit or feces). However, as demonstrated in previous studies, secondary transmission of HuNoV through environmental surfaces has emerged to be paramount due to the high virus titer shed by infected individuals, low infectious dose of the virus, and its long-term environmental persistence [3].

Disinfection is one of the key approaches recommended by the Centers of Disease Control and Prevention (CDC) to prevent and control HuNoV spreading from contaminated environmental surfaces. The U.S. Environmental Protection Agency (EPA) released a list of recommended disinfectants that should be used to disinfect HuNoV on non-porous surfaces [4]. Virus persistence and disinfection have been extensively studied using food contact surfaces and frequently touched surfaces in healthcare facilities, nursing homes, and schools. However, to our knowledge, there is no published study on the survival and disinfection of HuNoV on airplane cabin commonly touched surfaces despite the recent reports on HuNoV outbreaks during air travels. Holmes and Simmons [5] reported that one passenger, experiencing gastroenteritis, potentially infected 41 people, seated in the adjacent zones, on a 12.5-hour transpacific flight. Also, Kirking et al. [6] reported the transmission of HuNoV from 15 infected tour members to 7 other people in a flight from Boston, Massachusetts to Los Angeles, California [6]. The occurrence of HuNoV gastroenteritis event on board of airplanes suggests the aerosolization of the pathogen through projectile vomiting resulting in airborne contamination of surrounding surfaces and the potential for environmental transmission of HuNoV. This represents a significant public health problem since residual infectious virus on surfaces would result in recurrent, long-magnitude, and hard to control outbreaks.

Therefore, information on HuNoV persistence on airplane cabin commonly touched surfaces as well as the efficacy of disinfection is crucial. In this regard, this study aims to provide information about the survival of HuNoV on seat leather, plastic tray table, and seatbelt surfaces, and to study the efficacy of EPA registered disinfectants against HuNoV on those surfaces.

## 2. Materials and methods

### 2.1. HuNoV inoculum preparation

Dr. Jan Vinjé (CDC, Division of viral diseases, Atlanta, GA) kindly provided stool samples from a confirmed HuNoV GII.4 Sydney outbreak. A 20% suspension of the stool specimen was suspended in phosphate-buffered saline (PBS, pH 7.4), vortexed on high for 5 min, and then centrifuged at 4500 r.p.m. for 10 min. The supernatant (virus stock) was collected, aliquoted (500 µL), and stored at −80 °C. The final norovirus stock titer (9 log_10_ genomic copies/mL) was quantified by real-time RT-PCR.

Simulated gastric fluid (SGF) solution served as the additional organic load to mimic real life situation when vomit incident occurs in the airplane cabin. SGF suspension was prepared as described by Tung-Thomson et al. [7] The procedure could be simply described as following; deionized water was supplemented with 0.83% proteose peptone, 0.06% KH_2_PO_4_, 0.011% CaCl_2_, 0.01% lysozyme, 0.037% KCl, 0.005% bovine bile, 0.35% d-glucose, 0.00133% pepsin (all from Sigma-Aldrich Co. LLC., St. Louis, MO), and 0.205% NaCl (Fisher Scientific, Fair Lawn, NJ). The pH of the solution was adjusted to 2.5 using 1 M HCl. The solution was passed through a 0.2 µm cellulose membrane filter (VWR, Atlanta, GA) and stored at 4 °C. Fresh SGF was prepared for each experiment.

Fecal stocks with additional organic load (SGF) were prepared as follow: 1 mL HuNoV PBS stocks were concentrated to 200 µL using Amicon 30 KDa filters (Millipore, Ireland), 800 µL SGF was added, mixed thoroughly and stored at −80 °C until use.

### 2.2. Surface preparation and inoculation

Three commonly touched airplane cabin surfaces (seat leather, seat belt, and plastic tray table) were cut into 2.5 × 2.5 cm^2^ surface coupons. The surface coupons were sterilized by soaking in 10% bleach solution (30 min), then in 70% ethanol solution (30 min) and finally irradiated by UV light (overnight). Coupons were placed in sterile petri dishes, spot inoculated with 60 µL diluted (×10) HuNoV in PBS or SGF solutions. The inoculum titer was 6.0 × 10^6^ genomic copies/surface.

### 2.3. Survival study design

Coupons were allowed to dry in a biosafety hood for 2 h, then, they were placed in a controlled environmental chamber set at 21.8 °C and 26% relative humidity. Coupons were sampled at 0 h, 2 h, and then every 5 days for 30 days. HuNoV was eluted from surface using glycine (0.05 M)-saline (0.14 M) buffer (pH 8.5) as described [8] by pipetting repeatedly for 30 times. The procedure was carried out twice, and the total eluate volume (approximately 1 mL) was collected, concentrated to 200 µL using 30 Kda Amicon centrifugal filters (Millipore, Ireland) and stored at −80 °C until required for RNA extraction.

### 2.4. RNA extraction and RT-qPCR for HuNoV quantification

Prior to RNA extraction, norovirus surface eluates underwent an RNAase pretreatment step to degrade exposed RNA from partially destructed capsids or free floating viral RNA so only RNA from structurally intact capsids will be detected during RT-qPCR. This pre-treatment step has been used as an alternative method to distinguish between infectious and non-infectious virus particles preventing therefore an overestimation of the amount of infectious virus [9]–[11].

The RNAase pre-treatment was carried out as follow: Eluates (200 µL) were treated with 2 µL RNAse A (10 µg/mL) for 15 min at 37 °C. Samples were placed in the freezer (−20 °C) for 15 min to terminate the reaction. RNA extraction was performed using QIAamp Viral RNA Mini kit (Qiagen Sciences LLC, Louisville, KY) according to manufacturer's instructions. HuNoV RNA was recovered through two consecutive elutions using 40 µL of the Kit's elution buffer and immediately analyzed or stored at −80 °C until needed. RNA samples were tested by RT-qPCR for the detection and quantification of HuNoV by targeting the most conserved, sensitive and broadly reactive ORF1-ORF2 junctions in HuNoV genome using JJV2F and COG2R primers and probe RING2-TP (5-FAM-TGGGAGGGCGATCGCAATCT-BHQ-3)[12],[13]. The reaction mixture (final volume of 25 µL) consisted of 1.25 µL Primers JJV2F and COG2R (18 µM), 1.25 probe RING2-TP (5 µM), 12.45 µL nuclease free water, 6.3 µL TaqMan fast virus one step master mix (Life Technologies Corporation, Austin, TX), and 2.5 µL of the RNA template. The following amplification conditions were used: (i) reverse transcription for 10 min at 50 °C; reverse transcriptase inactivation/initial denaturation for 20 s at 95 °C and (iii) Amplification for 40 cycles of 3 s at 95 °C, 30 s at 60 °C.

HuNoV genomic copies were estimated by comparison with a standard curve established using RNA transcripts of the ORF1-ORF2 junction of the human norovirus genome (courtesy of Dr. Vinjé at the CDC, Atlanta, GA). The standard curve was established using the log_10_-transformed RNA genomic copies plotted against the threshold cycle (Ct) value (threshold 30) using linear regression.

### 2.5. Surface disinfection assays

For the disinfection of HuNoV contaminated surfaces, the CDC recommends the use of chlorine bleach solution with a concentration of 5% to 8% or other disinfectant registered as effective against norovirus by the EPA [14]. Since bleach based disinfectant cannot be used on board of airplanes because of the potential release of corrosive gases and VOCs [15], two anti-norovirus EPA registered disinfectants which can be used on airplane were chosen: hydrogen peroxide disinfectant cleaner (United States EPA registration No. 67619-24, hydrogen peroxide 1.4%, contact time 1 min), and broad-spectrum quaternary disinfectant cleaner (United States EPA registration No. EPA registration No. 67619-20, n-Alkyl (60% C14, 30% C16, 5% C12, 5% C18) dimethyl benzyl ammonium chlorides 0.105%, n-Alkyl (68% C12, 32% C14) dimethyl ethyl benzyl ammonium chlorides 0.105%, contact time 10 min). Clorox bleach germicidal cleaner (United States EPA registration No. 56392-7, sodium hypochlorite 0.65%, contact time 1 min) was chosen as a reference disinfectant for this study.

American Society for Testing and Materials (ASTM) method E1052-11 [16] was followed for disinfection suspension assays with minor modifications to account for reduced testing volume [10]: 60 µL HuNoV fecal suspensions (PBS or SGF) was suspended into 540 µL of each one of the disinfectants according to manufacturer's instructions. Disinfectant activity was neutralized by transferring 60 µL of HuNoV/disinfectant mixture into 540 µL 10% Dey/Engley neutralizing broth (D/E), (BD Difco, Sparks, MD). Positive controls (540 µL PBS added to HuNoV suspension) as well as neutralization control (540 µL neutralized disinfectant added to HuNoV suspension) were also conducted.

Surface assays were performed in accordance with ASTM method E1053-11 [17] with minor modifications: seat leather, seat belt, and plastic tray table coupons were inoculated with 60 µL HuNoV fecal suspensions (PBS or SGF), and allowed to dry for 2 h in a biosafety cabinet. Then, 540 µL of the disinfectants was pipetted onto coupons and allowed the appropriate contact time according to manufacturer's instructions. Afterwards, 5.4 mL of 10% Dey/Engley neutralizing broth (D/E), (BD Difco, Sparks, MD) was pipetted onto coupons for neutralization, and the coupon and all the liquid was transferred to a 50 mL conical tube, and vortexed for 30 s to eluate residual HuNoV. Positive and neutralization controls were also performed. Although the only available EPA disinfectants against norovirus are for non-porous surfaces, we decided to test them from a practical sense on porous surface (seat belt) to investigate their efficacy.

In the disinfection studies, HuNoV inactivation was calculated as previously described by subtracting log_10_ genomic copy number of treatment samples from the genomic copy number of the corresponding neutralization control sample [10]. In case of samples yielding non-detection via RT-qPCR analysis, HuNoV inactivation was calculated by subtracting the assay limit of detection value (1.18 log_10_ genome copies) from the corresponding log_10_ neutralization control values.

### 2.6. Statistical analysis

Both survival and disinfection studies were performed in two independent trials with duplicate surface coupons, and duplicate measurements. All data are reported as mean ± standard deviation. Statistical analysis was performed by one-way ANOVA followed by Tukey's HSD for pair-wise comparisons of means in JMP^®^, version Pro 13 (SAS Institute Inc., Cary, NC). A *p*-value of less than 0.05 was considered statistically significant.

## 3. Results

### 3.1. HuNoV surface recovery percentage

**Figure 1.**
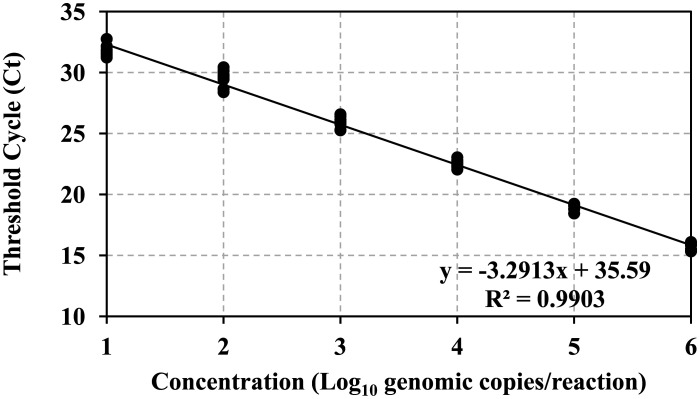
Human norovirus GII standard curve.

Three commonly touched airplane cabin surfaces (seat leather, seat belt, and plastic tray table) were artificially inoculated with HuNoV, with or without additional organic load (SGF and PBS, respectively). HuNoV genomic copies were estimated by comparison with a standard curve (Figure 1). Table 1 showed the percentage recovery of HuNoV from these three surfaces. Seat belt surface (porous/highly absorbent) had significantly less recovery percentage compared to plastic tray (non-porous) and seat leather which were non-absorbent (synthetic treated leather) (*p* < 0.05). Seat leather had similar HuNoV recovery percentage as the plastic tray table surface, and it was not considered porous (*p* > 0.05).

The standard curve was constructed using RT-qPCR-derived cycle threshold (Ct) values of 10-fold serially diluted GII.7 RNA transcripts. The log_10_ genomic copies were calculated per RT-qPCR reaction which contains 2.5 µL of template. For easy plotting, concentration 1 through 6 were used as x-axis as the solution volume (2.5 µL) was factored in when calculating the virus titer in all carried experiments.

### 3.2. Persistence of HuNoV on three commonly touched airplane cabin surfaces

**Figure 2.**
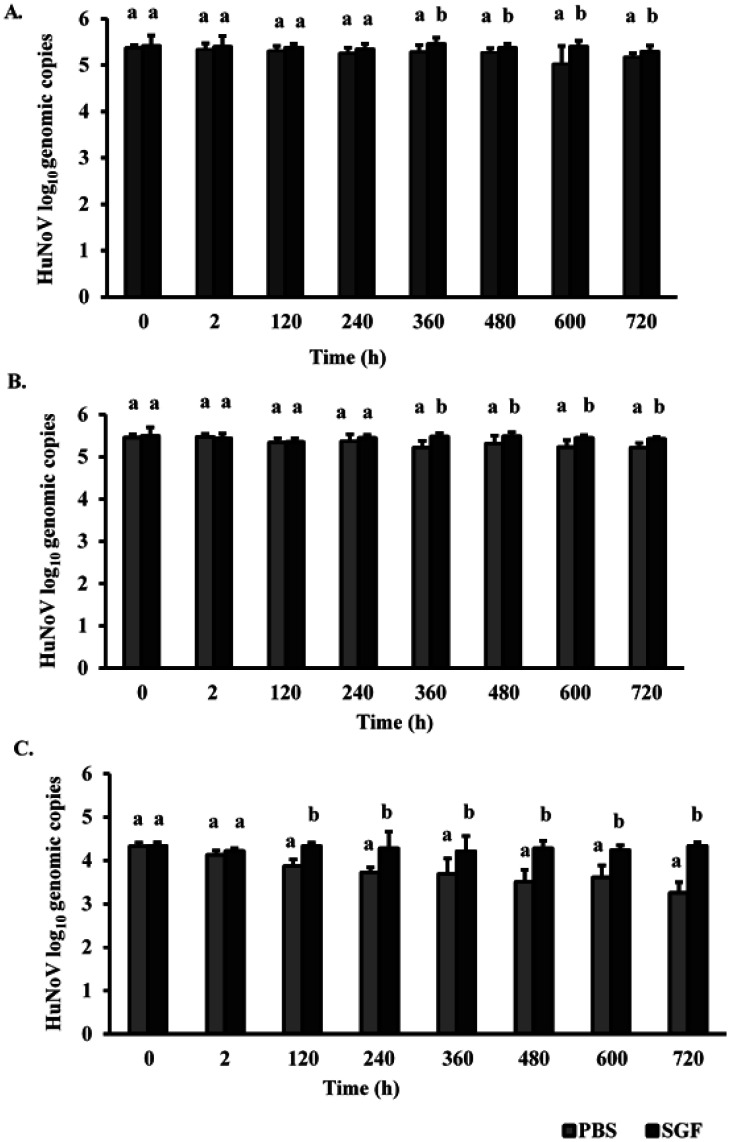
Survival of HuNoV on plastic tray (A), seat leather (B), and seat belt (C) coupons with (SGF) and without (PBS) additional organic load for 30 days. Experiments were performed in duplicates; all values represent mean ± standard deviation.

Persistence of HuNoV on three commonly touched airplane cabin surfaces (plastic tray table, seat leather, and seat belt) with or without the addition of organic load is shown in Figure 2. SGF was chosen as additional organic load in this study to mimic potential real-life scenario where airplane cabin surfaces can be soiled after a vomit incident. Results showed that HuNoV remained detectable throughout the 30-day persistence study period (720 h) on both non-porous (plastic tray and seat leather) and porous (seat belt) surfaces.

**Table 1.** HuNoV percentage recovery from artificially inoculated airplane cabin surfaces.

	HuNoV RE% (log recovery genomic copy)	
Surface Type	PBS	SGF
Seat belt	69.42 ± 0.49 ^a^	67.94 ± 0.55 ^a^
Plastic Tray	86.11 ± 0.54 ^b^	85.99 ± 2.74 ^b^
Seat Leather	87.56 ± 0.08 ^b^	87.20 ± 0.09 ^b^

Note: All values represented percentage recovery (log mean ± standard deviation). PBS denotes Phosphate-buffered saline. SGF denotes Simulated Gastric Fluid. Different letters show statistically significant differences (*p* < 0.05) when comparing percentage recovery between different surfaces within the same organic load (PBS or SGF).

PBS denotes Phosphate-buffered Saline. SGF denotes Simulated Gastric Fluid. Different letters show statistically significant differences (*p* < 0.05) when comparing log_10_ genomic copies by the effect of additional organic load at the same sampling time.

Additional organic load significantly influenced the survival of HuNoV. Statistical difference between PBS and SGF HuNoV samples was observed starting at day 5 in the case of seat belt surface, and at day 15 in the case of seat leather and plastic tray tables surfaces (*p* < 0.05). At day 30, significant higher survival of HuNoV was observed on all three surfaces when SGF was present.

### 3.3. Efficacy of anti-norovirus EPA registered disinfectants in HuNoV inactivation

**Figure 3.**
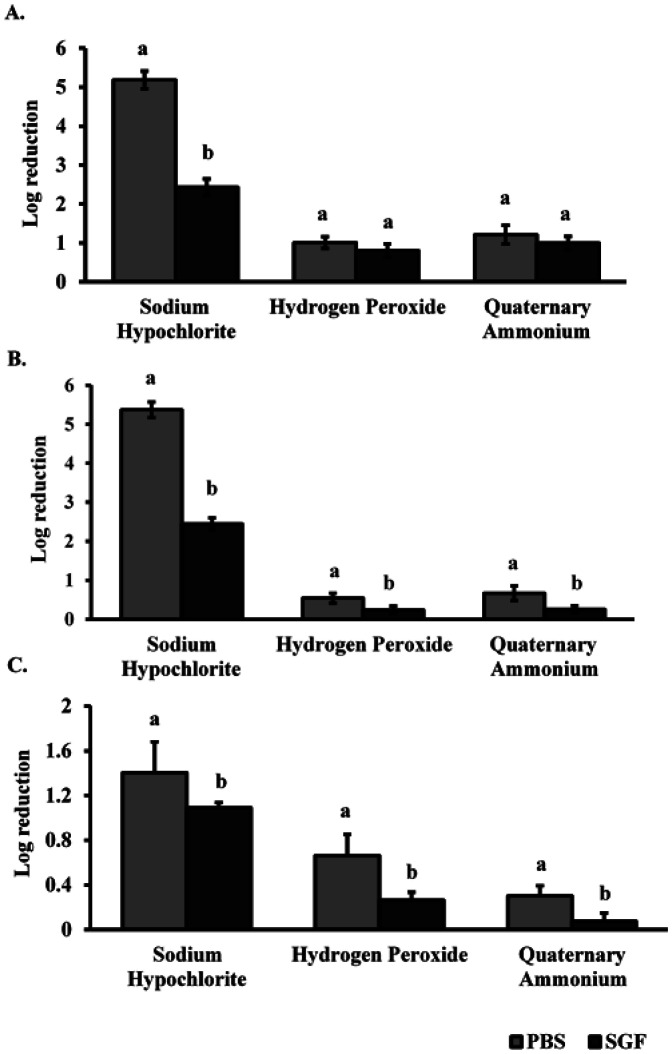
Inactivation of HuNoV without (PBS) and with (SGF) additional organic load following exposure to three anti-norovirus EPA registered disinfectants on plastic tray surface (A), seat leather (B), and seat belt (C).

The anti-noroviral efficacy of three EPA registered disinfectants (hydrogen peroxide, quaternary ammonium, and sodium hypochlorite) was tested on three commonly touched airplane cabin surfaces. Experiments were performed with and without additional organic load, simulated using SGF. Results were shown in Figure 3. Without SGF, on plastic tray seat and leather surfaces, only sodium hypochlorite which was used as a reference disinfectant in this study was efficient against norovirus resulting in 5.19 ± 0.22 and 5.38 ± 0.18 log_10_ reduction in HuNoV genomic copy number, respectively (Figure 3A,B). With SGF, significantly lower reductions in HuNoV genomic copies (2.43 ± 0.21 and 2.44 ± 0.14) were observed for these surfaces, respectively. Both hydrogen peroxide and quaternary ammonium-based disinfectants resulted in <1.25 log_10_ reduction in HuNoV genomic copy number regardless of the organic load (Figure 3A,B). For all three disinfectants, the lowest reductions were observed in the case of the seat belt surface where log reduction in HuNoV was <0.7 log_10_ HuNoV genomic copy number after hydrogen peroxide and quaternary ammonium treatments with SGF (Figure 3C).

Different letters show statistically significant differences (*p* < 0.05) when comparing log_10_ reduction in HuNoV genomic copies by suspension media (PBS or SGF) within the same disinfection treatment. The initial inoculum was ∼6 log_10_ genomic copies/surface.

## 4. Discussion

Contaminated surfaces have been well-documented to be a route of HuNoV transmission especially in enclosed settings such as long-term care facilities, hospitals, cruise ships, camping trips, and military settings [18]–[22]. Surfaces become contaminated by direct contact with contaminated gastrointestinal fluids, soiled hands, or aerosolized virus resulting from vomitus incidents [23]. The recent reports of HuNoV outbreaks on board of airplanes imply the contamination of airplane cabin surfaces with the pathogen, and them serving as HuNoV reservoir potentially leading to its secondary transmission through hand touching of contaminated surfaces. In fact, Barker et al. [24] showed that HuNoV could be transferred from contaminated surfaces to clean hands and then contaminated hands could transfer virus to a secondary surface, such as a phone or door handle [24]. Moreover, these authors reported that norovirus-contaminated hands could cross-contaminate a series of seven clean surfaces without additional recontamination of hands [24].

Since the ability of HuNoV to persist in the environment is one of the key factors in its transmission in any setting, the first objective of this study was to provide the crucial information on the survival of this pathogen using GII. 4 Sydney strain on three airplane cabin surfaces (plastic tray table, seat leather, and seat belt). Our findings revealed a high environmental stability of HuNoV on all three surfaces (porous and non-porous) where norovirus was detected until day 30 of the study (720 h). This is in accordance with previous studies which used different surfaces and desiccation conditions. For instance, Escudero et al. [8] reported the detection of HuNoV on three hard surfaces (stainless steel, ceramic, and Formica) for up to 42 days. Similarly, Lamhoujeb et al. [25] were able to detect HuNoV for up to 56 days on polyvinyl chloride and stainless steel. To the best of our knowledge, no other studies investigated the persistence of HuNoV on soft surfaces. There are currently few laboratory-based soft surfaces persistence studies which used feline calicivirus, murine norovirus, and MS2 bacteriophage as surrogates to study the survival of HuNoV on selected soft surfaces [26]–[29].

The observed protective effect of the additional organic load on the survival of HuNoV during desiccation was in accordance with previous studies which reported that a protective matrix was provided by organic matter to bacterial and virus pathogens enhancing therefore their environmental stability in different environmental conditions [23].

The second objective of this study was to investigate the efficacy of three anti-norovirus EPA registered disinfectants on the inactivation of HuNoV GII.4 Sydney on these same three commonly touched airplane cabin surfaces with the presence or absence of additional organic load. The disinfection was carried out after 2 h of surface inoculation with HuNoV. Since the survival study showed high environmental stability of HuNoV for all surfaces regardless of the presence or absence of the additional organic load, we believe that the observed viral reduction represents HuNoV inactivation resulting from exposure to disinfectants.

In this study, the bleach (sodium hypochlorite) was efficient in achieving the 4 log_10_ reduction required by EPA only at high concentration (6500 ppm), without additional organic load, and when used on non-porous surfaces. This is in accordance with a previous study by Park and Sobsey [30] who showed that even at high concentrations (5000 ppm), using sodium hypochlorite on fecally soiled stainless-steel coupons resulted in 1.4 log_10_ RNA genomic reduction in HuNoV after a contact time of 4 min [30].

Hydrogen peroxide and quaternary ammonium based disinfectants were not efficient in achieving the required 4 log_10_ EPA reduction regardless of the organic load. Girard et al. [31] reported that no reduction in HuNoV genomic copies were observed when applying quaternary ammonium products on stainless steel surfaces inoculated with HuNoV suspended in different buffers [31]. Gulati et al. [32] reported that hydrogen peroxide (11,000 ppm) was not effective when applied on stainless steel surfaces inoculated with feline calicivirus, a surrogate for HuNoV while a total inactivity of feline calicivirus was achieved with a contact time of 10 min [32].

It is important to mention that most disinfection studies use HuNoV surrogates such as murine norovirus and feline calicivirus with the latter being the EPA's choice for testing the anti-viral activity of disinfectants. Nevertheless, these cultivable surrogates may not always mimic the same behavior to disinfectants and inactivation methods as HuNoV [33],[34].

The low inactivation rate observed by all three disinfectants when additional organic load was present can be due to two key factors: the aggregation of virus particles on the surfaces and the protective effect provided by the organic load against disinfectants. In fact, it has been shown that on surfaces, viruses tend to aggregate contrary to being dispersed when in suspensions which make them more resistant to disinfection. In the core of an aggregate, factors such as disinfectant diffusion limitation as well as disinfectant degradation during its passage through the aggregate may prevent virus inactivation [35]. In addition, non-enveloped viruses' adhesion to solid surfaces promoted by hydrophobic and van der Waals interactions, as well as isoelectric point and ionic strength, was reported to decrease the access of the disinfectant to the virus [36].

Additionally, organic load has been shown to protect microorganisms from the effects of the disinfectant through physically blocking it from reaching the targeted microorganism, or through inducing chemical reaction with it, resulting in a lowered activity and efficacy [37]. Sodium hypochlorite is known to react with organic matter, which can diminish active concentrations as well as results in the formation of carcinogenic by-products, such as trihalomethanes [38].

Surface porosity seems to play a role in disinfectant efficacy as shown in this study. Log_10_ reduction in HuNoV genomic copy number after treatment of the contaminated seat belt surface with each of the three disinfectants was at its lowest compared to the other two surfaces, regardless the presence or absence of additional organic load. Sodium hypochlorite resulted in a reduction value <1.50 log_10_ in HuNoV genomic copy number reduction while hydrogen peroxide and quaternary ammonium yielded <0.3 log_10_ HuNoV genomic copy number reduction. Although these products are not intended for porous surfaces (there are none currently), we tried them out on the seatbelt surface for practical reasons: crew members could have these products at their disposition, and they can use them on all affected areas after a vomitus incidence.

Considering the effect of surface as well as organic load on the disinfection efficacy against HuNoV, higher disinfectant dose and longer contact time should be used when disinfecting microorganisms on surfaces [39],[40]. Also, based on our findings, we recommend the use of a cleaning step prior to disinfecting soiled surfaces in order to reduce the organic load on the surface and increase the disinfectant efficacy.

## References

[1] Chen SY, Chiu CH (2012). Worldwide molecular epidemiology of norovirus infection. Paediatr Int Child Health.

[2] Vinjé J (2015). Advances in laboratory methods for detection and typing of norovirus. J Clin Microbiol.

[3] Liu P, Chien YW, Papafragkou E (2009). Persistence of human noroviruses on food preparation surfaces and human hands. Food Environ Virol.

[4] United States Environmental Protection Agency (2018). List G: EPA's registered antimicrobial products effective against norovirus (Norwalk-like virus). https://www.epa.gov/sites/production/files/2018-04/documents/list_g_disinfectant_list_3_15_18.pdf.

[5] Holmes JD, Simmons GC (2009). Gastrointestinal illness associated with a long-haul flight. Epidemiol Infect.

[6] Kirking HL, Cortes J, Burrer S (2010). Likely transmission of norovirus on an airplane, October 2008. Clin Infect Dis.

[7] Tung-Thompson G, Gentry-Shields J, Fraser A (2015). Persistence of human norovirus RT-qPCR signals in simulated gastric fluid. Food Environ Virol.

[8] Escudero BI, Rawsthorne H, Gensel C (2012). Persistence and transferability of noroviruses on and between common surfaces and foods. J Food Prot.

[9] Knight A, Li D, Uyttendaele M (2013). A critical review of methods for detecting human noroviruses and predicting their infectivity. Crit Rev Microbiol.

[10] Moorman E, Montazeri N, Jaykus LA (2017). Efficacy of neutral electrolyzed water for inactivation of human norovirus. Appl Environ Microbiol.

[11] Topping JR, Schnerr H, Haines J (2009). Temperature inactivation of feline calicivirus vaccine strain FCV F-9 in comparison with human noroviruses using an RNA exposure assay and reverse transcribed quantitative real-time polymerase chain reaction—a novel method for predicting virus infectivity. J Virol Methods.

[12] Kageyama T, Kojima S, Shinohara M (2003). Broadly reactive and highly sensitive assay for Norwalk-like viruses based on real-time quantitative reverse transcription-PCR. J Clin Microbiol.

[13] Jothikumar N, Lowther JA, Henshilwood K (2005). Rapid and sensitive detection of noroviruses by using TaqMan-based one-step reverse transcription-PCR assays and application to naturally contaminated shellfish samples. Appl Environ Microbiol.

[14] Centers for Disease Control and Prevention (2018). Norovirus prevention. https://www.cdc.gov/norovirus/about/prevention.html.

[15] World Health Organization (2009). Guide to hygiene and sanitation in aviation. https://www.who.int/water_sanitation_health/hygiene/ships/guide_hygiene_sanitation_aviation_3_edition.pdf.

[16] American Society for Testing and Materials (2011). Standard test method to assess the activity of microbicides against viruses in suspension. E1052-11. https://www.astm.org/DATABASE.CART/HISTORICAL/E1052-11.htm.

[17] American Society for Testing and Materials (2011). Standard test method to assess virucidal activity of chemicals intended for disinfection of inanimate, nonporous environmental surfaces. E1053-11. https://www.astm.org/DATABASE.CART/HISTORICAL/E1053-11.htm.

[18] Isakbaeva ET, Widdowson MA, Beard RS (2005). Norovirus transmission on cruise ship. Emerging Infect Dis.

[19] Jones EL, Kramer A, Gaither M (2007). Role of fomite contamination during an outbreak of norovirus on houseboats. Int J Environ Health Res.

[20] Malek M, Barzilay E, Kramer A (2009). Outbreak of norovirus infection among river rafters associated with packaged delicatessen meat, Grand Canyon, 2005. Clin Infect Dis.

[21] Riddle MS, Smoak BL, Thornton SA (2006). Epidemic infectious gastrointestinal illness aboard US Navy ships deployed to the Middle East during peacetime operations—2000–2001. BMC Gastroenterol.

[22] Wu HM, Fornek M, Schwab KJ (2005). A norovirus outbreak at a long-term-care facility: the role of environmental surface contamination. Infect Control Hosp Epidemiol.

[23] Boone SA, Gerba CP (2007). Significance of fomites in the spread of respiratory and enteric viral disease. Appl Environ Microbiol.

[24] Barker J, Vipond IB, Bloomfield SF (2004). Effects of cleaning and disinfection in reducing the spread of norovirus contamination via environmental surfaces. J Hosp Infect.

[25] Lamhoujeb S, Fliss I, Ngazoa SE (2008). Evaluation of the persistence of infectious human noroviruses on food surfaces by using real-time nucleic acid sequence-based amplification. Appl Environ Microbiol.

[26] Buckley D, Fraser A, Huang G (2017). Recovery optimization and survival of the human norovirus surrogates feline calicivirus and murine norovirus on carpet. Appl Environ Microbiol.

[27] Fisher E, Shaffer R (2010). Survival of bacteriophage MS2 on filtering facepiece respirator coupons. Appl Biosaf.

[28] Lee JE, Zoh KD, Ko GP (2008). Inactivation and UV disinfection of murine norovirus with TiO2 under various environmental conditions. Appl Environ Microbiol.

[29] Yeargin T, Fraser A, Huang G (2015). Recovery and disinfection of two human norovirus surrogates, feline calicivirus and murine norovirus, from hard nonporous and soft porous surfaces. J Food Prot.

[30] Park GW, Sobsey MD (2011). Simultaneous comparison of murine norovirus, feline calicivirus, coliphage MS2, and GII. 4 norovirus to evaluate the efficacy of sodium hypochlorite against human norovirus on a fecally soiled stainless steel surface. Foodborne Pathog Dis.

[31] Girard M, Ngazoa S, Mattison K (2010). Attachment of noroviruses to stainless steel and their inactivation, using household disinfectants. J Food Prot.

[32] Gulati BR, Allwood PB, Hedberg CW (2001). Efficacy of commonly used disinfectants for the inactivation of calicivirus on strawberry, lettuce, and a food-contact surface. J Food Prot.

[33] Cromeans T, Park GW, Costantini V (2014). Comprehensive comparison of cultivable norovirus surrogates in response to different inactivation and disinfection treatments. Appl Environ Microbiol.

[34] Tung G, Macinga D, Arbogast J (2013). Efficacy of commonly used disinfectants for inactivation of human noroviruses and their surrogates. J Food Prot.

[35] Mattle MJ, Crouzy B, Brennecke M (2011). Impact of virus aggregation on inactivation by peracetic acid and implications for other disinfectants. Environ Sci Technol.

[36] Samandoulgou I, Fliss I, Jean J (2015). Zeta potential and aggregation of virus-like particle of human norovirus and feline calicivirus under different physicochemical conditions. Food Environ Virol.

[37] Meyer B, Morin VN, Rödger HJ (2010). Do European Standard Disinfectant tests truly simulate in-use microbial and organic soiling conditions on food preparation surfaces?. J Appl Microbiol.

[38] Di Cristo C, Esposito G, Leopardi A (2013). Modelling trihalomethanes formation in water supply systems. Environ Technol.

[39] Berg G, Chang SL, Harris EK (1964). Devitalization of microorganisms by iodine: I. Dynamics of the devitalization of enteroviruses by elemental iodine. Virology.

[40] Young DC, Sharp DG (1977). Poliovirus aggregates and their survival in water. Appl Environ Microbiol.

